# The RpfC (Rv1884) atomic structure shows high structural conservation within the resuscitation-promoting factor catalytic domain

**DOI:** 10.1107/S2053230X1401317X

**Published:** 2014-07-23

**Authors:** Francois-Xavier Chauviac, Giles Robertson, Doris H. X. Quay, Claire Bagnéris, Christian Dumas, Brian Henderson, John Ward, Nicholas H. Keep, Martin Cohen-Gonsaud

**Affiliations:** a Crystallography, Institute for Structural and Molecular Biology, Department of Biological Sciences, Birkbeck, University of London, Malet Street, London WC1E 7HX, England; b Centre de Biochimie Structurale, CNRS UMR 5048, 29 Rue de Navacelles, 34090 Montpellier, France; INSERM U1054, Université Montpellier I, Montpellier, France; c Department of Microbial Diseases, UCL–Eastman Dental Institute, University College London, 256 Gray’s Inn Road, London WC1X 8LD, England; d The Advanced Centre for Biochemical Engineering, Department of Biochemical Engineering, University College London, Torrington Place, London WC1E 7JE, England

**Keywords:** resuscitation-promoting factor, peptidoglycan, *Mycobacterium tuberculosis*

## Abstract

The first structure of the catalytic domain of RpfC (Rv1884), one of the resuscitation-promoting factors (RPFs) from *Mycobacterium tuberculosis*, is reported. The structure was solved using molecular replacement once the space group had been correctly identified as twinned *P*2_1_ rather than the apparent *C*222_1_ by searching for anomalous scattering sites in *P*1. The structure displays a very high degree of structural conservation with the previously published structures of the catalytic domains of RpfB (Rv1009) and RpfE (Rv2450). This structural conservation highlights the importance of the versatile domain composition of the RPF family.

## Abstract 2

The structure of the catalytic domain of RpfC (Rv1884) closely resembles those of RpfB and RpfE. This structural conservation highlights the importance of the versatile domain composition of the RPF family.

## 1. Introduction   

Resuscitation-promoting factors (RPFs) have attracted much interest since their discovery in the late 1990s. These proteins resuscitate bacteria that have entered a dormant state, allowing them to proliferate normally. Despite some key advances since the first protein identification and characterization, their precise mechanism of action remains elusive. The protein was first isolated in *Micrococcus luteus*, where a heat-labile, non-dialysable and trypsin-sensitive factor present in culture supernatants was able to resuscitate non-growing cells (Mukamolova *et al.*, 1998 ▶). The factor was identified as a protein and named resuscitation-promoting factor. In this same seminal study, corresponding genes in other GC-rich Gram-positive bacteria, most notably in *Mycobacterium tuberculosis*, were also identified. The resuscitating function was later confirmed in *M. tuberculosis* (Mukamolova *et al.*, 2002 ▶). This is an important finding, as one third of the human population is latently infected with *M. tuberculosis* in a dormant form. This represents a large population reservoir for reactivation of tuberculosis and also a potential novel therapeutic avenue for treating tuberculosis.

Sequence analysis coupled with homology modelling led to the hypothesis that the conserved RPF catalytic domain could be a transglycosidase belonging to the family of c-type lysozymes (Cohen-Gonsaud, Keep *et al.*, 2004 ▶). The prediction was confirmed by the first solution structure of the RpfB catalytic domain from *M. tuberculosis*, which showed that the domain is a short version of the c-type lysozyme lacking the first helix (Cohen-Gonsaud *et al.*, 2005 ▶). Later, various experiments unambiguously demonstrated that the RPF domain is a peptidoglycan hydrolase (Mukamolova *et al.*, 2006 ▶; Telkov *et al.*, 2006 ▶).

Five RPF paralogues are present in *M. tuberculosis* (*rpfA–E*). They contain a conserved catalytic domain, but the domain composition shows variability also found in other species (Ravagnani *et al.*, 2005 ▶). The mycobacterial RPF proteins share a common 70-amino-acid RPF domain and the presence of N-terminal signal sequences suggesting that the proteins are translocated to an extracellular location. RpfC (176 amino acids), RpfD (154 amino acids) and RpfE (172 amino acids) consist almost solely of the RPF domain and signal sequence and are supposed to have a paracrine function. RpfB (362 amino acids) possesses a G5 domain that may be involved in peptidoglycan binding (Ruggiero *et al.*, 2009 ▶) and a prokaryotic membrane lipoprotein lipid-attachment site that may confer it with a juxtacrine function, while RpfA (407 amino acids) possesses a low compositional complexity domain that may confer an autocrine function (Mukamolova *et al.*, 1998 ▶).

Initial studies showed that deletion of individual *rpf* genes had no significant phenotypic consequences (Downing *et al.*, 2004 ▶; Tufariello *et al.*, 2004 ▶). This suggests that the mycobacterial RPF proteins are functionally redundant. The deletion of the entire mycobacterial *rpf* gene family is also dispensable for growth (Kana *et al.*, 2008 ▶). However, phenotypic alterations appear with the deletion of three or more *rpf* genes and reveal a functional hierarchy of the mycobacterial Rpf proteins that has been reviewed elsewhere (Kana & Mizrahi, 2010 ▶).

The question arises as to the functional specificity of the various RPF paralogues. Is specificity based on small changes within the RPF catalytic domain structure itself or on the domain organization? The solution structure (Cohen-Gonsaud *et al.*, 2005 ▶) and various X-ray structures of RpfB (Ruggiero *et al.*, 2009 ▶, 2013 ▶; Squeglia *et al.*, 2013 ▶) and, very recently, the structure of RpfE have been published (Mavrici *et al.*, 2014 ▶). In this paper, we describe the X-ray structure of the RpfC catalytic domain. Despite the presence of multiple copies in the asymmetric unit, twinning and strong noncrystallographic translation, we succeeded in solving the structure using molecular replacement. The structure highlights the high degree of structural conservation within the RPF domains, which could explain why the mycobacterial paralogues are functionally redundant.

## 2. Methods   

### 2.1. Protein preparation and crystallogenesis   

The sequence coding for the catalytic domain of RpfC (residues Gly68–Lys159 of UniProt RPFC_MYCTU) was cloned into the *Nde*I and *Nhe*I sites of the pET15-TEV plasmid to generate a recombinant protein containing a six-histidine tag at the N-terminus cleavable by *Tobacco Etch Virus* (TEV) protease (Cohen-Gonsaud, Barthe *et al.*, 2004 ▶). The N-terminus after cleavage corresponds to the first amino acid of the mature RpfC after predicted cleavage of the signal peptide. The experimentally determined start codon is residue 34 of the UniProt entry (RPFC_MYCTU; Raman *et al.*, 2004 ▶) and the first 34 residues (34–67 of the UniProt entry) are the signal peptide. Therefore, we number the protein structure from residue Gly1, which is Gly68 in the UniProt entry. The last 17 residues of the protein were predicted to be disordered from the RpfB structure and were excluded from this construct.

Protein expression was carried out in *Escherichia coli* Rosetta2 (DE3) strain grown in ZYM5052 auto-induction medium at 25°C for 36 h (Studier, 2005 ▶). Cells were harvested and lysed by sonication in 100 m*M* Tris pH 7.5, 2 m*M* β-mercaptoethanol (BME) (buffer *A*). The lysate was cleared by centrifugation at 48 000*g* for 1 h at 4°C. The supernatant was loaded onto a nickel–NTA column (GE Healthcare) equilibrated with buffer *A* and was eluted with buffer *A* supplemented with 300 m*M* imidazole (buffer *B*). The eluted protein fraction was dialysed (3 kDa cutoff) against 20 m*M* Tris pH 7.5, 2 m*M* BME (buffer *C*) overnight at 4°C in the presence of TEV protease. The cleaved protein was further purified by gel filtration on a HiLoad Superdex 75 column (GE Healthcare, Amersham, England) equilibrated in buffer *C* before being concentrated for crystallization trials. Crystals grew readily in 22 of the 96 conditions of The Classics Suite (Qiagen, Hilden, Germany), but all belonged to the same space group, with the condition 0.1 *M* sodium citrate pH 5, 20%(*w*/*v*) PEG 6000 giving the best crystals. Some optimization of this condition was carried out and a slight improvement was achieved using 0.1 *M* sodium citrate pH 5, 22%(*w*/*v*) PEG 6000. The crystals were cryoprotected in the crystallization condition with 20% ethylene glycol.

### 2.2. Data collection, processing and phasing   

Default processing of data sets using either *XDS* (Kabsch, 2010 ▶) or *iMosflm* (Powell *et al.*, 2013 ▶) always gave space group *C*222_1_. Data sets were reprocessed in *P*2_1_ (Table 1 ▶) with care taken to use an *R*_free_ selection that meant that all pseudoequivalent reflections were in the refined or the free data set. A thin-shell *R*_free_ file was obtained using *SFTOOLS* from *CCP*4 (Winn *et al.*, 2011 ▶) from an RpfC data set indexed in *C*222_1_ with unit-cell parameters *a* = 65.12, *b* = 142.88, *c* = 88.93 Å, α = β = γ = 90°. The initial file was expanded to the lowest symmetry space group *P*1. From there, the file was modified to match the unit-cell parameters to the integrated *P*2_1_ data. The first re­indexing was carried out to set the angle to β = 114° using the transformation matrix (100, 001, −110) with unit-cell parameters *a* = 65.12, *b* = 88.93, *c* = 157.02 Å. Finally, the software *REINDEX* from *CCP*4 was used with settings *h* = *h*, *k* = *k*, *l* = *l*/2 to give the correct unit-cell lengths *a* = 65.12, *b* = 88.93, *c* = 78.51 Å, α = γ = 90, β = 114.50°. The free set was then reduced to the *P*2_1_ asymmetric unit and used as the source of free reflection flags for all other data sets.

Initial phasing was carried out by *MrBUMP* (Keegan & Winn, 2008 ▶) using the crystal structure of the catalytic domain of RpfB (PDB entry 3e05; Ruggiero *et al.*, 2009 ▶). A solution with four copies in the asymmetric unit was found in *C*222_1_ but would not refine below an *R*_free_ of 0.500 using *MOLREP* (Vagin & Teplyakov, 2010 ▶). However, two copies of this model were found in the *P*2_1_ unit cell and refined with the use of twinning to a final *R*_free_ of 0.236 using *REFMAC*5 (Murshudov *et al.*, 2011 ▶; see Table 2 ▶). There is a noncrystallographic translation of (0.554, 0.0, 0.109) in fractional coordinates of 50% of the origin peak. With the improvements in molecular replacement including noncrystallographic translation since this work was originally carried out, current versions of *Phaser* (McCoy *et al.*, 2007 ▶) and *MOLREP* can solve this structure more routinely from a single RpfB chain.

## 3. Results and discussion   

### 3.1. Structure-solution problems   

Many data sets were collected from crystals of RpfC or the point mutations RpfC_E13A or RpfC_E13M with and without potential substrates and including selenomethionine-substituted RpfC_E13M at the ESRF, SLS, SOLEIL and Diamond synchrotrons. The automatic space-group assignment for all data sets gave the space group as *C*222_1_, with unit-cell parameters of around *a* = 66, *b* = 141, *c* = 90 Å, α = β = γ = 90°. The resolutions of the data sets ranged from 3.0 to 1.9 Å. This would predict four copies of the RpfC chain in the asymmetric unit. We failed to obtain a molecular-replacement solution using our NMR structure (PDB entry 1xsf; Cohen-Gonsaud *et al.*, 2005 ▶). Slightly better solutions were found using the crystal structure of the RpfB catalytic domain, with *R* and *R*_free_ of around 0.45 and 0.50, respectively, but these would not refine further. Attempts at Se or S SAD also did not give solutions. However, anomalous site searching using charge flipping (Dumas & van der Lee, 2008 ▶), which works in *P*1, indicated that the data were probably in space group *P*2_1_, as eight sites could be found using the SeMet RpfC_E13M data in this space group. This data set did not yield a useable map, probably owing to the data set being twinned (0.41 from a Britton plot) and the presence of only weak anomalous signal that only extended to around 3.8 Å as assessed by *phenix.xtriage* (Zwart *et al.*, 2005 ▶) and *CTRUNCATE* from *CCP*4. However, molecular replacement with the *C*222_1_ solution from the crystal structure of RpfB (Ruggiero *et al.*, 2009 ▶) in space group *P*2_1_ (unit-cell parameters *a* = 65, *b* = 88, *c* = 78 Å, α = γ = 90, β = 114.50°) to give eight copies in the asymmetric unit and refining with twin operators 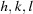 and 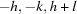 allowed refinement to acceptable *R* and *R*_free_ values on carefully selecting the free set (see Table 2 ▶). Twinning was not apparent from the *L*-test (Yeates, 1988 ▶) or the moments of *E*, but was estimated for the final data set as 0.41 from the *H*-test (Padilla & Yeates, 2003 ▶) and 0.45 in a Britton plot (Fisher & Sweet, 1980 ▶) as tested by *CTRUNCATE*. Other data sets gave similar twinning. The final refined twinning fraction in *REFMAC*5 for the deposited structure was 0.463 for 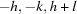. Despite soaking and co-crystallizing with a range of substrates and substrate fragments, for example *N*-acetyl­glucosamine (NAG), polymers of up to five repeats of *N*-acetylglucosamine and NAG-*N*-acetylmuramic acid, and peptido­glycan fragments that are generated by a number of enzymes, we never obtained clear density for substrates in the active site. We have therefore deposited the structure of the wild-type RpfC catalytic domain (PDB entry 4ow1).

### 3.2. Structure analysis   

**Figure 1:**
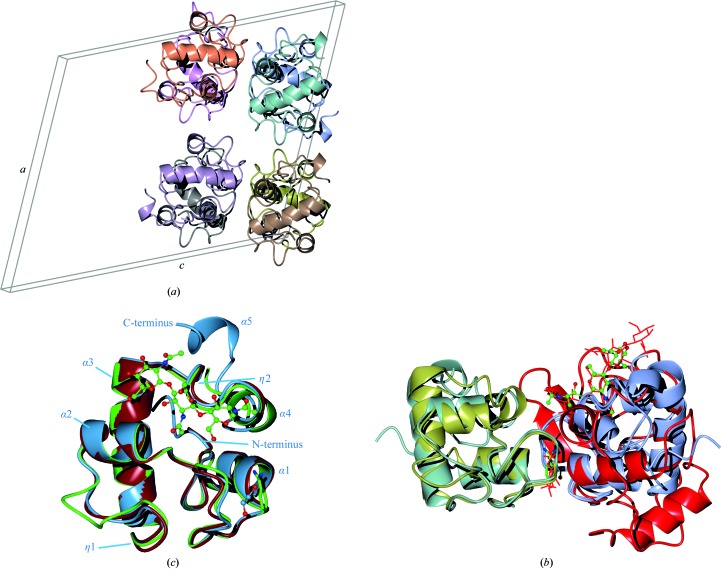
Structure of RpfC compared with RpfB and RpfE. (*a*) RpfC asymmetric unit and cell edges looking at the *ac* plane. The noncrystallographic translation of (0.554, 0.0, 0.109) can be seen. (*b*) Superposition of RpfB [green; PDB entry 4kpm chain *A* with tri-*N*-acetylglucosamine (NAG)_3_ and benzamidine], RpfC (light blue; PDB entry 4ow1 chain *A* with ethylene glycol) and RpfE (tan; PDB entry 4cge chain *A*) with the small insertion at the bottom left of RpfB. (*c*) Comparison of RpfB [PDB entry 4kpm; chain *A*, ice blue; chain *B*, gold; (NAG)_3_ and benzamidine in green ball-and-stick representation] with RpfC (PDB entry 4ow1; chain *A*, ice blue; chain *T*, cyan; ethylene glycol, light blue) and lysozyme [PDB entry 1lzs; chain *A*, red; (NAG)_4_ and (NAG)_2_ shown with fat red bonds]. This shows the conservation of a crystallographic interface between RpfB and RpfC and the overlap of the ethylene glycol and benzamidine sites. All images were produced with *CCP*4*mg* (McNicholas *et al.*, 2011 ▶).

The asymmetric unit consists of eight copies of the RpfC chain. A set of four copies is generated by two twofold axes perpendicular to the crystallographic twofold; a single translation of (0.554, 0.0, 0.109) then generates the second set of four copies (Fig. 1 ▶ *a*). Coupled with twinning the two folds give rise to the pseudo-*C*222_1_ symmetry.

Chains *A*, *E* and *S* have the most residues modelled into electron density (Gly1–Lys86) with an extra helix beyond the end of the conserved domain (Gly78). Chain *B* has the least modelled residues (Pro4–Gly78); the other chains are between these limits. We have modelled an ethylene glycol (the cryoprotectant) where a benzamidine molecule is present in the RpfB structures with PDB codes 4kpm (Squeglia *et al.*, 2013 ▶) and 4emn (Ruggiero *et al.*, 2013 ▶). As for the benzamidine in 4kpm, this is only seen in one of the similar interfaces. Benzamidine and ethylene glycol are not all that similar, but this observation indicates that this region in RPFs prefers binding small organic molecules to water. This region is part of the predicted binding site of a hexasaccharide based on superposition of the lysozyme-cleaved hexasaccharide complex with PDB code 1lzs (Song *et al.*, 1994 ▶). The crystal packing of the two adjacent chains close to the benzamidine/ethylene glycol site is almost perfectly conserved in our RpfC structure and in the RpfB structures, despite there being no evidence of this contact being physiological. The two pairs of chain superimpose with an r.m.s.d. of 1.1 Å over 149 residues using *SSM* (Krissinel & Henrick, 2004 ▶), which is not much larger than that for the single chains (see below). The RPF domains are sufficiently close to clash with the superposed disaccharide in this region. The trisaccharide in 4kpm coincides with the other part of the cleaved saccharide in 1lzs (Fig. 1 ▶ *b*).

**Figure 2:**
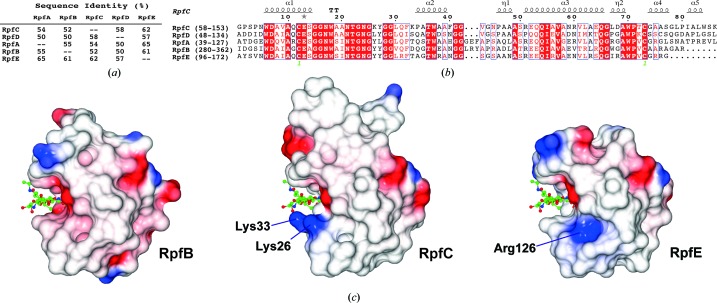
Sequence and charge variation and conservation. (*a*) Sequence identity between the five *M. tuberculosis* RPF domains calculated using *MUSCLE* (Edgar, 2004 ▶). (*b*) Alignment of the RPF domains of RpfA, RpfB, RpfC, RpfD and RpfE. The number ranges in the names correspond to the UniProt entry. The numbering along the sequence is that of the mature RpfC after signal sequence cleavage and also of the PDB entry. (*c*) Electrostatic surfaces of RpfB, RpfC and RpfE showing the variation in electrostatics around the saccharide-binding cleft. The triNAG of RpfB from the structure superposition is shown in all three images and the images are from the same viewpoint.

As expected, the structural conservation between the new RpfC catalytic domain structure that we have determined in this study and the extensively studied RpfB domain is high. The calculated C^α^ r.m.s.d. between the two structures (our structure *versus* PDB entry 4kl7; Squeglia *et al.*, 2013 ▶) is only 0.90 Å for 76 residues aligned by *SSM* with 52% sequence identity over the domain (Figs. 2 ▶ *a* and 2 ▶ *b*). Compared with the recent RpfE structure (PDB entry 4cge; Mavrici *et al.*, 2014 ▶), the calculated C^α^ r.m.s.d. is even lower at 0.82 Å for 77 residues with 62% sequence identity (Figs. 1 ▶ *c* and 2 ▶ *a*). Most of the backbone geometry is conserved, including the connecting loops between the helices. This is in accordance with the first NMR structure that we determined, where the 30 calculated structures shared a low r.m.s.d. of 0.57 Å, low thermal motion as shown by NOE (Nuclear Overhauser Effect) ratios (Cohen-Gonsaud, Barthe *et al.*, 2004 ▶) and a well ordered fold for the RPF domain. The only difference observed is located within a short sequence insertion that is present in the RpfB RPF domain compared with the other four *M. tuberculosis* RPF proteins (Figs. 1 ▶ *c* and 2 ▶ *b*). In RpfC two residues display an elongated conformation (^42^GVGN^45^), very similar to RpfE (^137^GSGS^140^), to connect α-helices 2 and 3, while a 3_10_-helix (^321^GLRYAPR^327^) is present in RpfB. This small change within the secondary-structure composition does not change the relative orientation of α-helices 2 and 3 within the RPF fold (Fig. 1 ▶ *c*). The variation in surface charge between RpfB and RpfE has previously been noted (Mavrici *et al.*, 2014 ▶). RpfC has two lysines, Lys26 and Lys33, on one side of the sugar-binding cleft, which are tyrosines in RpfA, RpfB and RpfD or a leucine in RpfE and serine or threonine in RpfA, RpfD and RpfE or an aspartate in RpfB (Fig. 2 ▶ *b*), respectively. This leads to a different charge distribution around the ligand-binding pocket, which may have a role in specificity (Fig. 2 ▶ *c*). Mavrici *et al.* (2014 ▶) suggested that Arg126 may play a role in binding the peptide part of the peptidoglycan, conferring specificity on RpfE.

## 4. Conclusion   

The RpfC structure catalytic domain displays a high degree of structural conservation with the other members of the mycobacterial resuscitation-promoting factor family. Based on the structure that we have solved, we propose that the five RPFs from *M. tuberculosis* have similar substrates, although variation in charge around the active site may give rise to small variations in the specificity for different peptidoglycan modifications. The high degree of conservation of the RPF domain explains why the protein is functionally redundant, but most importantly shows that the auxiliary domain composition is mainly responsible for the functional variability.

## Supplementary Material

PDB reference: RpfC, 4ow1

## Figures and Tables

**Table 1:** Data collection and processing Values in parentheses are for the outer shell.

Diffraction source	ESRF beamline ID23-1
Wavelength ()	1.0723
Temperature (C)	173
Detector	ADSC Quantum 315r CCD
Crystal-to-detector distance (mm)	216.5
Rotation range per image ()	0.35
Total rotation range ()	210
Space group	*P*2_1_
*a*, *b*, *c* ()	66.23, 89.93, 78.09
, , ()	90, 115.08, 90
Mosaicity ()	0.259
Resolution range ()	44.971.90 (1.941.90)
Total No. of reflections	266668 (17421)
No. of unique reflections	64809 (4200)
Completeness (%)	99.6 (100)
Multiplicity	4.1 (4.1)
*I*/(*I*)	8.3 (2.6)
*R*_r.i.m._ †	0.087 (0.439)
Overall *B* factor from Wilson plot (^2^)	18.1

† Estimated *R*_r.i.m._ = *R*_merge_[*N*/(*N* 1)]^1/2^, where *N* is the data multiplicity.

**Table 2:** Structure solution and refinement Values in parentheses are for the outer shell.

Resolution range ()	38.421.90 (1.9491.900)
Completeness (%)	99.6
Cutoff	0
Twin fractions ( 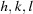 )/( 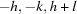 )	0.537/0.463
No. of reflections, working set	61793 (4462)
No. of reflections, test set	3271 (271)
Final *R*_cryst_	0.205 (0.216)
Final *R*_free_	0.236 (0.261)
ESU based on free *R*	0.027
No. of non-H atoms	
Protein	4762
Ligand	0
Solvent (including EDO)	240
Total	5002
R.m.s. deviations	
Bonds ()	0.022
Angles ()	1.823
Average *B* factors (^2^)	
Protein	31.7
Ligand	0
Water	27.3
Ramachandran plot	
Most favoured (%)	98.2
Allowed (%)	1.8
